# A feasibility study of a novel low-level light therapy for digital ulcers in systemic sclerosis

**DOI:** 10.1080/09546634.2018.1484875

**Published:** 2018-07-31

**Authors:** M. Hughes, T. Moore, J. Manning, J. Wilkinson, S. Watson, P. Samraj, G. Dinsdale, C. Roberts, L. E. Rhodes, A. L. Herrick, A. Murray

**Affiliations:** a Centre for Musculoskeletal Research, The University of Manchester, Salford Royal NHS Foundation Trust, Manchester Academic Health Science Centre, Manchester, UK;; b Department of Rheumatology, Salford Royal NHS Foundation Trust, Salford, UK;; c Research and Development, Salford Royal NHS Foundation Trust, Salford, UK;; d Medical Physics Department and University of Manchester, Manchester Academic Health Science Centre, Salford Royal NHS Foundation Trust, UK;; e Medical Physics Department, Salford Royal NHS Foundation Trust, UK;; f Centre for Biostatistics, Institute of Population Health, School of Medicine, The University of Manchester, Manchester, UK;; g Photobiology Unit, Dermatology Centre, Division of Musculoskeletal and Dermatological Sciences, The University of Manchester, Salford Royal NHS Foundation Trust, Manchester Academic Health Science Centre, Manchester, UK;; h NIHR Manchester Musculoskeletal Biomedical Research Centre, Central Manchester NHS Foundation Trust, Manchester Academic Health Science Centre, UK;; i Photon Science Institute, The University of Manchester, UK

**Keywords:** Systemic sclerosis, scleroderma, digital ulcers, phototherapy

## Abstract

**Background:** Locally acting, well-tolerated treatments for systemic sclerosis (SSc) digital ulcers (DUs) are needed.

**Objectives:** Our primary aim was to investigate the safety, feasibility, and tolerability of a novel low-level light therapy (LTTT). A secondary aim was to tentatively assess efficacy.

**Methods:** A custom-built device comprising infrared (850 nm), red (660 nm), and violet (405 nm) LEDs was utilized. DUs were irradiated with 10 J/cm^2^ twice weekly for 3 weeks, with follow-up at weeks 4 and 8. Any safety concerns were documented. Patient opinion on time to deliver, feasibility, and pain visual analogue score (VAS; 0–100, 100 most severe) was collected. Patient and clinician DU global assessment VAS were documented. DUs were evaluated by laser Doppler perfusion imaging pre- and post-irradiation.

**Results:** In all, 14 DUs in eight patients received a total of 46 light exposures, with no safety concerns. All patients considered LTTT ‘took just the right amount of time’ and was ‘feasible’, with a low associated mean pain VAS of 1.6 (SD: 5.2). Patient and clinician global DC VAS improved during the study (mean change: –7.1 and –5.2, respectively, both *p* < .001). DU perfusion significantly increased post-irradiation.

**Conclusions:** LTTT for DUs is safe, feasible, and well tolerated. There was an early tentative suggestion of treatment efficacy.

## Introduction

Systemic sclerosis (SSc) is a complex autoimmune connective tissue disease characterized by microvascular abnormalities, fibrosis of the skin and internal organs, and immune system activation (1). Digital ulcers (DUs) are common in patients with SSc and are responsible for much of the pain and disability associated with the disease (2–7). Half of patients with SSc report a history of DUs, often occurring early in the course of the disease (7–9). DUs, in particular those located on the fingertip, are believed to be ischemic in etiology (7,10,11). Patients with SSc also commonly have marked finger contractures, which may predispose to recurrent trauma and can make wound care challenging for patients. Despite there being treatments available to prevent DUs (12,13), recurrent ulceration remains a major source of morbidity in some patients with SSc. Furthermore, DUs are often superficially infected, in particular, with *Staphylococcus aureus* (14) and can undergo deeper bony progression (7). Unfortunately, despite targeted intervention, in some patients, digital amputation may be necessary for refractory DUs (15).

Current drug therapies (e.g. intravenous prostanoids) (16,17) used to treat existing DUs, tend to rely upon systemic vasodilation (with the aim to increase perfusion to the DU). These treatments are therefore often poorly tolerated, leading to dose reduction and/or discontinuation. Hence, there is a strong therapeutic rationale to develop ‘locally’ acting treatments for DUs, which would likely be well tolerated by patients (i.e. without systemic vasodilation) and could potentially avoid the need for hospitalization to administer intravenous therapies.

Low-level light therapy (LLLT) is an area of growing clinical interest. While its use has been largely empirical and complicated by the application of various wavelengths and dosimetric parameters, it is now reported in a number of studies (albeit with a lack of any high-quality randomized controlled trials) to be a safe and effective treatment for refractory skin (diabetic, pressure, and venous) ulcers (18–27). The majority of previous studies have reported that LLLT was associated with around an additional 50% (range of 30–60%) (18,19,21–24,26,27) in improvement in ulcer status compared with the comparator group (conventional wound care and/or placebo light treatment).

Light treatment within the red and near-infrared spectrum is believed to stimulate a wide number of cellular processes (often referred to as ‘biostimulation’) which are thought to benefit wound healing, including (but not limited to) stimulation of fibroblast and macrophage number and function, increasing leucocyte mobility, modulation of growth factors and inflammatory mediators, and by promoting collagen deposition and neovascularization (28,29).

Infrared light is also associated with ambient heating and an increase in blood flow (although this is likely short-lived), and improved tissue oxygenation. Red light can also have an antimicrobial effect through excitation of naturally occurring porphyrins (30). In a blinded, randomized, placebo-controlled, single treatment trial, photodynamic therapy with red light and an exogenous photosensitizer caused a significant reduction in bacterial load of diabetic ulcers, and a trend toward ulcer healing (31).

Blue light also has an antibacterial effect including activity *in vitro* against *Staphylococcus aureus* (32). Impact of the LLLT may occur both via effects on the ulcer bed and on the ulcer margins, including with respect to bacteria present. While blue light can reach bacteria residing on the surface or within the epidermis, bacteria can also colonize deeper dermal components of the skin, and blue light will be less effective than red/infrared in reaching these. DUs in patients with SSc are relatively superficial, with an average depth of 1 mm (as measured by high-frequency ultrasound); therefore, this is unlikely to be an important disadvantage (33). While there is much less of a precedent for the use of violet (or blue) light to treat ulcers, it is important to consider that blue light is more photochemically active than red light and causes more reactive oxygen species generation (34). Blue light has been shown to increase perfusion through stimulation of local nitric oxide (NO) release, with relaxation of vascular smooth muscle, and to increase wound healing in a skin excision model (35,36).

Against this background, the primary aim of the study was to assess the safety, feasibility, and tolerability of a novel light treatment, combining infrared, red, and violet wavelengths, for DUs in patients with SSc. The rationale for choosing these wavelengths was to improve DU healing as described above, including via the mechanisms implicated in biostimulation (e.g. collagen production), through an increase in DU perfusion, and with a potential additional antimicrobial effect. Our secondary aim was to tentatively assess whether this light therapy might have a beneficial effect on DU healing: first, by patient and clinician opinion and independent assessment of photographic record, and second, by measuring perfusion as assessed by laser Doppler imaging (LDI).

## Materials and methods

Patients >18 years of age with SSc-spectrum disorders (mainly SSc) were recruited into the study. Eight patients (demographic and clinical characteristics are summarized in Table 1) were recruited into the study, one of whom was studied on three occasions (re-entered the study twice with new DUs). In all, 10 ‘sets’ of treatment were undertaken. A total of 14 DUs were treated. Two DUs were treated at the same time in four patients. The majority of patients (*n* = 7) had SSc: four with limited and three with diffuse cutaneous disease (37). One patient had an SSc-spectrum disorder, the clinical features in whom included Raynaud's phenomenon with abnormal nailfold capillaroscopy. Participant progression throughout the study is presented in Figure 2. Patients with serious infection (e.g. osteomyelitis) of the DU were excluded. Patients were recruited between 20 January 2015 and 15 January 2016, and the last study visit was 18 April 2016. The study was approved by NRES Committee North West-Liverpool Central (REC reference: 14/NW/1400), and all patients gave written consent. Treatment study visits were conducted in a temperature-controlled laboratory at 23 °C. ClinicalTrials.gov identifier: NCT02472743.

**Table 1.** Characteristics of the patients who participated in the study.

Age (mean, SD) (years)	48.5 (15.2)
Sex (female:male)	7:1
RP duration (mean, SD) (years)	16.1 (11.7)
Disease duration, from first non-RP clinical manifestation) (mean, SD) (years)	11.9 (7.6)
Presence of significant finger contractures	2
SSc-associated autoantibodies (*n*)	
Anticentromere	3
Anti-Scl-70	2
Anti-RNA polymerase III	1
History of digital vascular disease	
History of intravenous vasodilator therapy	6
History of debridement	3
Organ complications (*n*)	
Pulmonary fibrosis	2
Pulmonary hypertension	1
Drug treatment (*n*)	
Vasodilatory therapy	7
Calcium channel blocker	3
Phosphodiesterase type-5 inhibitor	3
Endothelial receptor antagonist	2

### Visit protocol

Patients with DUs were prospectively recruited at Salford Royal NHS Foundation Trust (SRFT), Manchester, UK: a tertiary referral center for SSc, either at their routine clinic attendance or during an episode of hospitalization. We included DUs located at both the fingertip and extensor aspect of the hands, as well as other sites on the hands. Two DUs on the same hand were included if they could both be illuminated within the light treatment area. The majority of patients were receiving treatment with vasodilatory drug therapies (Table 1). Patients attended a total of eight study visits each over 2 months. Light treatment was administered twice weekly for 3 weeks (i.e. the first six study visits) with follow-up visits at week 4 (visit 7) and week 8 (visit 8). Patients were asked to abstain from caffeine-containing drinks and from smoking (as these both cause vasoconstriction) for at least 4 h prior to attending each study visit. Sterile gauze and/or water could be used (based upon the clinical judgment of the operator), using gloves, to clean the surface of the DU of any debris, which could potentially interfere with the light treatment. Patient baseline demographics and clinical characteristics were collected. At each study visit, any changes to drug treatment were documented, as well as DU visual analogue score (VAS) (patient and clinician opinion) as described below. At each *treatment* study visit, LDI was performed to include DU, immediately before and after light exposure. At each study visit, a clinical photograph of the DU was taken, which was graded by an independent assessor at a later date (described below). Our treatment protocol is described immediately below.

### Light-based treatment

**Figure 1.**
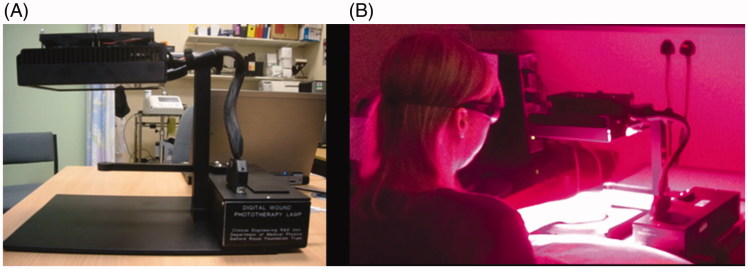
Light-based treatment for SSc-related DUs. A: Side profile of the light device. B: The light device in operation treating an extensor aspect DU in a patient with SSc. The distance from the panel of LEDs to the treatment area is approximately 15 cm.

A custom-built light treatment device (Figure 1) was designed and constructed in-house at SRFT. This comprised 32 light-emitting diodes (LEDs): 10 infrared (850 nm, LZ1-00R400, LED Engin Inc.), 11 red (660 nm, LZ1-00R200, LED Engin Inc.), and 11 violet (405 nm, LZ1-00UA00, LED Engin Inc.). The respective peak wavelength (bandwidth) (full-width half-maximum (FWHM)) was 860 (bandwidth 36) nm, 663 (20) nm, and 406 (18) nm. The mean irradiances in the treatment plane were 8.2 mW/cm^2^, 9.1 mW/cm^2^, 10.4 mW/cm^2^ for the infrared, red, and violet LEDs, respectively. All wavelengths were given simultaneously, with a total dose of 10 J/cm^2^ at each exposure. The rationale for the dosimetry was its broad similarity to that used in previous LLLT studies of other types of ulcers (21,23–26,30). The configuration of the device ensured that the LED outputs spatially blended such as to prevent any ‘hotspots’/’coldspots’. Before each treatment, there was an initial period of calibration at each wavelength with an in-built optical power meter and software, to ensure that the administered dose was within 10% of the intended dose. Each exposure took approximately 15 min. The specified irradiation times (variation in applied dose and time of application is <5%) based on measured irradiances are 19.6, 17.8 and 15.8 min for the infrared, red, and violet light, respectively.

**Figure 2.**
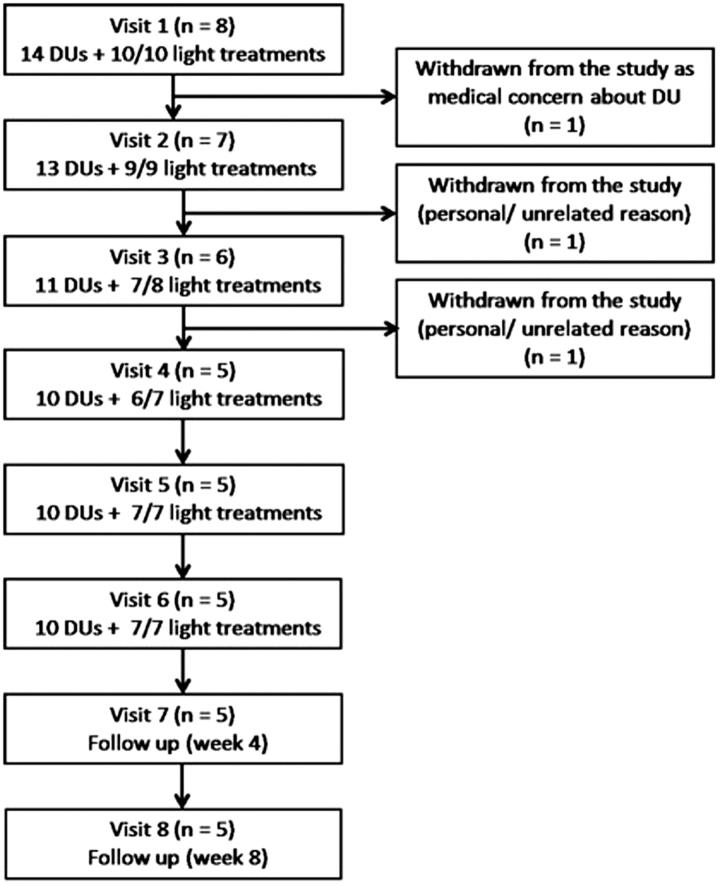
Patient progression throughout the study. The number of administered (and scheduled) light treatments is indicated for each study visit. There were two occasions on which the light treatment could not be administered due to technical failure of the device, and this was early in the course of the study: patient 1 visit 4 and patient 2: visit 3 (who was no longer studied after this visit).

The operator had full command of the light device through a custom-built control interface on an attached computer. The device provided ±10% of the specified irradiance within a 10 cm diameter circle (78 cm^2^). Therefore, more than one DU could be treated simultaneously per hand, and allowed some freedom of movement for the patient for comfort. Preliminary data in four healthy controls (not included in this manuscript) indicated that the change (increase) in skin temperature as measured by an attached thermocouple was in the range of 2.5 °C–5.8 °C using 405 nm light (by a prototype lamp with identical LEDs used in this study) at irradiance values of 50 mW/cm^2^. We applied 10 J/cm^2^ in approximately 200 s and measured the skin temperature within the irradiated zone at the reference site. There was heterogeneity in response: in two subjects, the increase rapidly decayed to baseline after the irradiance finished, whereas, in two subjects, it again decayed but did not reach baseline, staying 0.5–1.0 °C higher for >30 min post-exposure. It is not clear what the contribution to the observed temperature rise is from a photothermal effect (i.e. direct conversion of the absorbed optical radiation to heat), or by the production of photochemical vasodilators.

Possible optical radiation hazards were assessed to verify that neither patient nor operator would be exposed to intensities exceeding those set in the UK Control of Artificial Optical Radiation at Work Regulations 2010. Nonetheless, both patient and operator wore goggles at all times the light device was in operation, as the light was slightly uncomfortable to directly observe. There was no contact between the DUs and the LEDs (Figure 1). Before and after each patient contact, the light device treatment area was cleaned with an alcohol-based wipe.

### Outcome measures

#### Assessment of safety, feasibility, and tolerability

At each treatment study visit, any safety issues were documented (e.g. new infection of the DU). Patient opinion on the time taken to administer (‘too little time’, ‘just the right amount of time’, ‘too long’) and overall feasibility (‘not feasible’, ‘indifferent’, ‘feasible’) of light treatment was collected. Patient-reported pain on a VAS scale (0–100, 100 being the most severe) associated with the light treatment was recorded, to assess tolerability.

#### Patient and clinician DU assessment

Two experienced clinicians (MH and AH) with an interest in SSc and digital vascular disease performed the clinician-based assessments. At each study visit (before the light treatment was administered), patient and clinician (MH) global assessments of the DU were independently performed on a VAS (0–100, 100 being most severe). In addition, a clinical photograph of the DU was obtained by a professional medical photographer, including a small (1 cm) length scale in close proximity to the DU, to give an indication of the size of the DU. Patients with ≥1 DU were asked to rate each DU separately, as were clinicians in these cases. For each DU, an independent assessor (AH) compared the baseline photograph with the subsequent study visit photographs. The assessor was only aware of the time point of the baseline photograph, and all the others were provided in a random order. Only the photographs of the second treatment visit per week (i.e. visits 2, 4, and 6) and both follow-up visits (7 and 8) were compared to baseline. The perceived change in DU appearance was graded on a Likert scale ranging from –2 (much worse) to +2 (much better).

#### LDI (visits 1–6)

LDI measures blood flow, which allows a perfusion map (in arbitrary units) to be produced. LDI was performed immediately before and after the light exposures at the site of the DU(s). We used a MoorLDI-vr (Moor Instruments, UK) laser Doppler imager (red, 633 nm). Perfusion measurements from the LDI images were assessed using a standard region of interest (ROI) for the ‘DUCore’ (center of the DU) and the ‘DUPeriphery’ (tissue immediately adjacent to the DU). Relative hyperemia of the skin immediately surrounding the ischemic DU center has been described (10,11). Therefore, we chose to study both of these regions as they may be important in the DU healing of SSc-DUs.

### Statistical analysis

Descriptive statistics are provided for the safety, feasibility, and tolerability data. Individual patients (*n* = 8, one studied on three occasions) and DUs (*n* = 14) were considered as unique entities in the analyses. A linear mixed-effects model was used to assess change in DU status for both patient and clinician opinion (including the independent assessment of photographs) and LDI. This approach accounts for the correlation of repeated measurements on a single individual and gives the rate of change across the study period (i.e. from one visit to the next). For LDI, we added a pre/post-treatment indicator variable, to assess the improvement immediately following treatment. LDI data were log-transformed for the analysis. A *p* values of <.05 was considered as statistically significant. All analyses on the data were performed using STATA version 13 (StataCorp, USA).

## Results

### DU disease

We treated similar numbers of fingertip (*n* = 4), extensor (*n* = 5), and lateral aspect of the finger (*n* = 5) DUs. None of the DUs had any significant crust/eschar present, or were subject to debridement. In addition, one patient commenced intravenous iloprost before the third light treatment for DU disease, and went on to receive all the intended six light treatments as scheduled.

### Outcome measures

#### Assessment of safety, feasibility, and tolerability

A total of 46 light treatments were successfully administered and no safety concerns were encountered during the course of the study. All the participants considered that the light treatment (*n* = 45, data not available for one study visit) took ‘just the right amount of time’ and was ‘feasible’. Patient-reported pain associated with light treatment (*n* = 45 sessions, data not available for one study visit) was low with a mean VAS score of 1.6 (SD 5.2), with the majority of sessions (*n* = 40) being considered completely painless (VAS score 0).

#### Patient and clinician DU assessment

**Figure 3.**
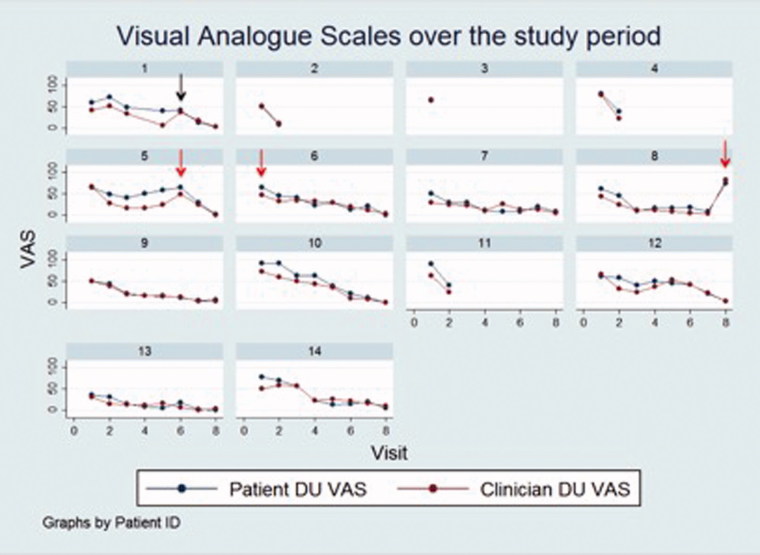
Patient and clinician assessment of the 14 SSc-DUs. For individual DUs, both the patient and clinician VAS are presented according to study visit. Black arrow (top row): The patient reported trauma to the DU (DU 1) before the study visit. Red arrows (middle row): Antibiotic therapy. Antibiotic therapy was prescribed prior to study entry in one patient (DU 6), and after light therapy was completed, and during follow-up, in two patients, respectively (DUs 5 and 8).

**Figure 4.**
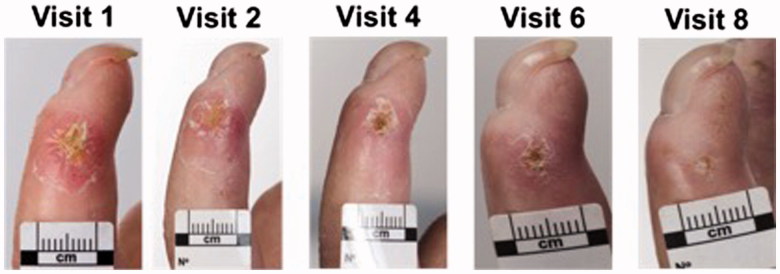
Clinical photographs are presented for a patient with SSc with a lateral aspect DU. There was an improvement in both patient and clinician DU opinion (Figure 3, DU 14) and independent clinician opinion (data not shown). A clinical photograph could not be performed at week 7 (visit 7) due to operational issues.

Figure 3 depicts both the patient and clinician DU VAS over the course of the study for each of the 14 ulcers. The mean (SD) patient and clinician DU global assessment VAS at baseline were 64.0 (16.1) and 53.8 (14.8), respectively, and at the end of the study period were 10.7 (22.7) and 11.3 (25.3), respectively. There was a trend for improvement in both patient and clinician VAS score over the study period; patient DU VAS improved by –7.1 (95% CI: –8.6 to –5.7) units at each visit (*p* < .001) and for clinicians by –5.2 (95% CI: –6.5 to –3.8) (*p* < .001). Compared to baseline, the mean reduction (SD) in DU VAS at the end of the six light treatments (week 3, visit 6) for patients was 58.5% (29.3%) and for clinicians was 57.7% (26.0%). At the final study visit (week 8, visit 8), the reduction in DU VAS compared to baseline for patients was 82.8% (37.6%) and for clinicians was 74.9% (57.4%). The mean (SD) perceived change in DU appearance as assessed by photographs improved by 0.14 (95% CI: 0.0–0.3) (*p* = .01) units per week compared to baseline. An example of serial DU clinical photographs is presented in Figure 4.

#### LDI

For the analysis of LDI measurements, 37 (pre-treatment) and 32 (post-treatment) LDI measurements were available for both the DUCore and DUPeriphery sites. There was a significant increase in the relative (compared to baseline) mean perfusion after light treatment of 0.32 (95% CI: 0.13–0.52) (*p* = .0013) at the DUCore and of 0.15 (95% CI: 0.0–0.30) (*p* = .04) at the DUPeriphery.

## Discussion

The key findings of our study are that our novel light therapy is a feasible and well-tolerated treatment for DUs showing short-term safety. We successfully administered the light treatment to DUs located on a variety of locations on the hands, including in patients with marked finger contractures. Furthermore, to our knowledge, this is the first study to examine a light-based treatment for DUs in patients with SSc.

Although it was not the primary intention of our study to examine treatment efficacy, there was an early suggestion that this therapy may have a beneficial effect on DU healing although we accept that this was a small study without a control group. Throughout the study, there was a significant improvement in DU status, including the independent grading of photographs by a blinded assessor. Of interest, over half the improvement in DUs from baseline was observed during the treatment period (i.e. within 3 weeks), as assessed both by patient and clinician opinion (58.5% and 57.7%, respectively). In addition, as a possible ‘local’ therapy (to improve perfusion to ‘ischemic’ DUs to improve healing), our light treatment was indeed associated with a significant increase in DU perfusion, in particular at the ischemic center.

Previous studies of LLLT for common types of cutaneous ulcers have investigated red and infrared (either broadband or combined) light, and have usually utilized either LED-based or low-level laser systems. It remains controversial whether there is any difference in possible efficacy between these two different types of light sources. We chose to develop a LED-based system for several reasons including (but not limited to) that LEDs are readily available in a range of wavelengths, relatively inexpensive compared to lasers, and future treatment devices (which potentially could be used by patients at home) would be safer, smaller, more portable, and cheaper using LEDs.

The wavelengths chosen of red and infrared light are comparable to those used in previous LLLT studies, and the violet light utilized likely has antibacterial action against *Staphylococcus aureus*. In our study, we exploited naturally occurring photosensitizing agents (e.g. porphyrins and potentially other chromophores). Photochemical (similar to previous studies in diabetic, pressure, and venous ulcers) and photothermal effects could both be very beneficial in the healing of ischemic SSc-related DUs. An effect on NO production would be expected with violet light as it is observed to be enhanced by both blue and ultraviolet A therapy/ultraviolet B therapy (UVA)/UVB wavelengths. Marmalis et al. (38) demonstrated that blue light inhibits fibroblast proliferation, and therefore theoretically could improve DU healing by reducing associated skin thickening.

Several studies have reported that psoralen and ultraviolet A therapy (PUVA) and UVA1 phototherapy have beneficial effects in SSc including on skin sclerosis and DUs (39). Inoue et al. (40) reported the successful use of PUVA in a patient with diffuse cutaneous SSc and progressive DU disease. PUVA therapy was administered three times weekly for a month (cumulative dose: 23 J/cm^2^) and was associated with improvement in DUs, as well as in skin sclerosis and revascularization, both as assessed by skin biopsies. Although PUVA might be effective in SSc-related DU, it would be less convenient as a treatment than LLLT because it involves giving a photosensitizer, as well as UV radiation.

All the patients included in our study had previous DUs, and the majority had previously received intravenous prostanoid therapy for DU disease. In addition, most were receiving vasoactive therapy for digital vascular disease, with several patients prescribed ‘advanced’ therapies (phosphodiesterase inhibitors or endothelin receptor antagonists). Therefore, our patients are likely to be representative of those patients with SSc with the greatest clinical need for the development of light-based treatment for DU disease.

Our study has a number of important considerations. Although the number of studied patients (and DUs) was relatively small, in this feasibility study we have successfully addressed the safety, feasibility, and tolerability of LLLT for SSc-DUs. This was an open study, and although there was a suggestion of treatment efficacy, this needs to be confirmed through the scientific rigor of a double-blind, randomized, placebo-controlled trial. Our study benefited from a robust study design including (but not limited to) the independent (blinded) grading of clinical photographs and measurement of DU perfusion by LDI. We adopted a pragmatic approach and allowed one patient to re-enter the study twice with new DUs. One patient commenced the systemic vasodilator iloprost during the study due to evolving medical concerns about the severity of the DU, but given this was a primarily a feasibility study, she continued in this. The wavelengths and treatment frequency may potentially be optimized in future research. In addition, we used a simple grading system for patients and clinicians to grade DUs, and for an independent (blinded) assessor to grade photographs of DUs. In future studies, other outcome measures of efficacy may be utilized (e.g. an independent clinician physically assessing the DUs) as well as objective ulcer measurement, and microbial load, pre- and post-treatment.

## Conclusions

In conclusion, our targeted novel LLLT was safe, feasible, and well tolerated by patients. Combining infrared, red, and violet light has the potential advantage of activating a wide range of beneficial photochemical effects. In addition, there was an early suggestion of treatment efficacy, with a significant improvement in DUs during the course of the study. The local increase in DU perfusion with light treatment may obviate the systemic vasodilation inherent in most current treatment approaches to SSc-related DU. Future research is warranted to develop our targeted light-based treatment as a locally acting therapy for DUs in patients with SSc, with further clinical potential to apply the technology for the treatment of a wider range of ulcers.
